# New approaches to management of neonatal hypoglycemia

**DOI:** 10.1186/s40748-016-0031-z

**Published:** 2016-05-10

**Authors:** Paul J. Rozance, William W. Hay

**Affiliations:** Perinatal Research Center, Department of Pediatrics, University of Colorado School of Medicine, 13243 E 23rd Ave, MS F441, Aurora, CO 80045 USA

**Keywords:** Neonatal hypoglycemia, Dextrose gel, Continuous glucose monitoring, Infant of a diabetic mother, Late preterm, Small for gestational age, Large for gestational age, Intrauterine growth restriction

## Abstract

Despite being a very common problem after birth, consensus on how to manage low glucose concentrations in the first 48 h of life has been difficult to establish and remains a debated issue. One of the reasons for this is that few studies have provided the type of data needed to establish a definitive approach agreed upon by all. However, some recent publications have provided much needed primary data to inform this debate. These publications have focused on aspects of managing low blood glucose concentrations in the patients most at-risk for asymptomatic hypoglycemia—those born late-preterm, large for gestational age, small for gestational age, or growth restricted, and those born following a pregnancy complicated by diabetes mellitus. The goal of this review is to discuss specific aspects of this new research. First, we focus on promising new data testing the role of buccal dextrose gel in the management of asymptomatic neonatal hypoglycemia. Second, we highlight some of the clinical implications of a large, prospective study documenting the association of specific glycemic patterns with neurodevelopmental outcomes at two years of age.

## Background

Hypoglycemia is one of the most frequently encountered problems in the first 48 h of life, and low glucose concentrations are perhaps the most common biochemical abnormality seen by providers caring for newborns. Unfortunately, the optimal strategy for managing this problem remains elusive and is a matter of differing interpretations of the available literature [1–8]. New data to inform the optimal management of these newborns is urgently needed [9]. Especially controversial is the management of asymptomatic but at-risk newborns, most commonly those with a history or physical exam consistent with being born late-preterm, large for gestational age (LGA), small for gestational age (SGA), or growth restricted, or an infant of a diabetic mother (IDM). The reason for this controversy is that numerous studies have shown that, with the exception of the LGA group, these newborns have worse neurodevelopmental outcomes than healthy term babies [10–13] and that in some of these groups, worse neurodevelopmental outcomes are associated with the presence of neonatal hypoglycemia [12]. To date, no study has shown that preventing or treating the hypoglycemia in these groups leads to better outcomes, making it uncertain whether hypoglycemia has a causal role in producing the worse outcomes. In fact, the statement made by the AAP in 1993 remains accurate today, “… there is no evidence that asymptomatic hypoglycemic infants will benefit from treatment [14].” The goal of this review is to discuss recent primary research that has added important new data to consider when devising strategies to manage this group of newborns. These recent data hold promise for optimizing our approach to managing neonatal hypoglycemia, especially in the at-risk groups noted above.

## Asymptomatic neonatal hypoglycemia

The newborns most at risk for, and most frequently screened for, asymptomatic hypoglycemia include late preterm, LGA, SGA, and/or intrauterine growth restricted (IUGR) infants, and IDMs [4]. Frequent milk feedings with repeated glucose measurements is the current standard treatment for asymptomatic hypoglycemia in these groups of patients [4]. This approach allows mothers and babies to remain together, provides nutrient substrates to support gluconeogenesis as it develops, and ensures that the hypoglycemia resolves. If hypoglycemia persists despite frequent milk feedings, a continuous intravenous dextrose infusion may be indicated. The following approach to the rate of dextrose infusion for asymptomatic hypoglycemia can be considered. A dextrose infusion rate of 3–5 mg/kg/min can be used for IDMs, as this avoids overstimulation of insulin secretion and accounts for the larger fat mass that these infants have. A dextrose infusion rate of 4–7 mg/kg/min can be used for most term and near term infants. A dextrose infusion rate of 6–8 mg/kg/min often is necessary in IUGR infants. This accounts for their greater brain/body weight ratio and physiological observations made in animal models of IUGR of both increased peripheral insulin sensitivity and increased insulin secretion as their postnatal physiology is normalized and insulin-suppressive catecholamine secretion is reduced [15–20]. Glucose concentrations must be followed closely as some of these IUGR infants, especially those very preterm, also can have hyperglycemia, due to reduced insulin secretion capacity, diminished muscle mass for glucose disposal, and persistent glucose production [17, 18, 20–30]. A continuous intravenous dextrose infusion, usually preceded by an intravenous dextrose bolus (200 mg/kg given over 5 min), also is indicated if these newborns develop symptomatic hypoglycemia. In fact, partial or complete resolution of the symptoms with correction of glucose concentrations is considered proof that the symptoms were caused by the low glucose concentrations [31]. Intravenous dextrose infusions, however, are not benign; they cause discomfort and stress due to the placement of an intravenous catheter, admission to a NICU, and physical separation of the mother and newborn which risks impairing the timely and successful establishment of breastfeeding and bonding. Preventing these complications of intravenous dextrose infusions, while safely managing asymptomatic low glucose concentrations, has many potential benefits.

## Dextrose gel

To this end, Harris, et al. undertook a large, randomized, placebo-controlled, double-blinded study of buccal dextrose gel for the treatment of asymptomatic hypoglycemia, defined as a plasma glucose less than 47 mg/dL (2.6 mmol/L) irrespective of postnatal age [32]. The dextrose gel (200 mg/kg) or placebo gel was massaged into the infant’s dried buccal mucosa and the infant was encouraged to feed. If the baby still had a low glucose concentration 30 min after gel administration, or if the baby developed recurrent hypoglycemia, the treatment with study gel continued for a total of six doses over 48 h. The specific characteristics of the populations studied included late preterm (35–36 weeks gestational age), LGA (>90th percentile or >4500 g), SGA/IUGR (<10th percentile or <2500 g), and IDM newborns. In these groups dextrose gel decreased the number of episodes of hypoglycemia, decreased the recurrence rate of hypoglycemia, increased exclusive breastfeeding rates at discharge, and decreased the need for admission to the neonatal intensive care (NICU) unit to treat hypoglycemia. The number of newborns needed to treat to prevent one admission to the NICU for hypoglycemia was only eight. It is important to note that in this study the newborns, regardless of randomization, were still managed with aggressive oral feeding (mostly breastmilk) to treat hypoglycemia. There were no adverse events reported and continuous interstitial glucose monitoring (to which the care providers were blinded) did not identify more frequent, clinically unrecognized episodes of rebound or recurrent hypoglycemia in the dextrose gel group, thereby establishing short term safety. Of note, overall admission rates to the NICU for all causes were not statistically significantly different between groups (38 % in the dextrose gel group vs. 46 % with placebo gel). The most likely cause of the lack of effect on overall NICU admission rates, despite the reduction in NICU admissions to treat hypoglycemia, is simply that the sample size was too small to show a difference in this secondary outcome.

Despite these encouraging short term results, there was some concern that the dextrose gel treatment might have adversely impacted long term neurodevelopmental outcomes. Reasons for concern included a potential delay in definitive treatment with intravenous dextrose, rapid overcorrection and iatrogenic hyperglycemia, and increased variability in glucose concentrations [33, 34]. Information from continuous glucose monitoring sensors (CGMS), which was blinded to the caregivers, was reassuring regarding these concerns. When the researchers looked at the CGMS data, however, they found that the time to achieve an interstitial glucose concentration greater than 47 mg/dL (2.6 mmol/L) with dextrose gel was as rapid as has been reported for correction with intravenous dextrose given at 8 mg/kg/min--about twenty minutes [35]. CGMS data also showed that rebound hypoglycemia was rare in both groups and the incidence of recurrent hypoglycemia was less in the dextrose gel group. While these data support the safety and efficacy of he dextrose gel established by intermittent glucose sampling, CGMS was only used in a subset of the patients in this study. Therefore, it is important that two year outcomes have recently been published [36]. 78 % of the original hypoglycemic cohort was available for assessment of outcomes at two years of age. Fortunately, there were no differences between the dextrose gel group and placebo group for neurosensory impairment, processing difficulties, or secondary growth and developmental outcomes. The high rates of abnormal outcomes in both groups is concerning and should prompt further research into optimal management of these patients [36].

When considering the adoption of dextrose gel into clinical practice, it must be remembered that although the study was quite large and included a broad representation of the main at-risk groups, it was a single center study. A multi-centered trial confirming these results would broaden the applicability of this therapy. This issue is highlighted by the results for improved rates of exclusive breast feeding at two weeks of age [32]. The rates of exclusive breastfeeding at this age are quite high in this New Zealand population. Whether this benefit would be replicated in populations with higher or lower rates of breast feeding is unknown. However, if a new trial included hospitals without a NICU, the benefits of dextrose gel might also include lower transfer rates of these patients to hospitals that have intensive care capabilities. It also is important to note that the definition of hypoglycemia and treatment cut-off were both 47 mg/dL (2.6 mmole/L), irrespective of gestational and postnatal age. This definition does not take into account the age related changes in mean and lower limits of normal glucose concentrations that occur in the first days of life [37]. The definition of hypoglycemia, screening frequency, and screening duration also may vary from what is used in some clinical practices [4, 8]. Clinicians should ensure that they have accounted for these differences when considering adoption of dextrose gel into clinical practice. Not only was screening for hypoglycemia quite frequent, in this study the clinicians used a very reliable point of care device. This device uses the gold standard glucose oxidase method for measurement of plasma glucose concentrations, which is much more accurate than bedside glucometers [38]. Glucose concentrations were measured at one hour of age, then before feeds every 3–4 h for the first 24 h of age, and then before feeds every 6–8 h for the next 24 h. This protocol identified 46 % of the enrolled patients as hypoglycemic, who were included in the study. This relatively high incidence of neonatal hypoglycemia in this population likely is due to the rigorous and frequent screening protocol, the method used to measure glucose concentrations, the higher glucose concentration threshold that was used to define hypoglycemia compared to other published guidelines [4], and a definition of hypoglycemia that does not exclude infants who have low glucose concentrations during the normal physiological nadir that occurs in normal infants in the first hours of life [7, 37].

Other important considerations are that the dextrose gel treatment was tested in infants who were at-risk for hypoglycemia but were otherwise well-appearing and asymptomatic [32]. The results cannot be applied to infants with severe, symptomatic, and recurrent hypoglycemia, and/or if the infant is not in one of the at-risk groups studied. Furthermore, the dextrose gel did not eliminate the need for intravenous dextrose therapy. Over 10 % of infants treated with dextrose gel in this study had an episode of rebound hypoglycemia and over 20 % had an episode of recurrent hypoglycemia after a documented normal glucose concentration. While these rates were similar to or better than those in the placebo group, practitioners should not be using this therapy without close follow up of subsequent glucose concentrations, clinical signs of hypoglycemia, and documentation of resolution of the hypoglycemia [4, 8]. Despite these limitations, the therapy appears safe and effective at preventing NICU admissions for hypoglycemia and it may be appropriate for some hospitals to adopt into clinical practice. Indeed, such efforts are already being described with promising results [39, 40]. Making guidelines for managing low glucose concentrations in the first days of life safe and easy to follow, while at the same time promoting increased maternal-infant interactions and increased breastfeeding rates, is critical for all practitioners caring for newborns. Buccal dextrose gel appears to have a promising role in achieving these goals and caregivers can be more confident that the early benefits of dextrose gel are not associated with worse two year neurodevelopmental outcomes [36].

## Continuous glucose monitoring

Another interesting feature of the dextrose gel study is the use of CGMS to continuously monitor interstitial glucose concentrations. One important observation is that many episodes of hypoglycemia, documented by both blood obtained per their low glucose concentration screening protocol and CGMS measurements, resolved spontaneously, and were not associated with bedside nursing observations of clinical signs that might be interpreted as symptoms of hypoglycemia. Thus, while CGMS remains a research tool, this observation demonstrates the potential for CGMS to reduce unnecessary treatment for hypoglycemia. For example, CGMS may identify patients in whom their low glucose concentrations have resolved prior to commencement of interventions. However, there also is the potential for CGMS to increase unnecessary treatment for hypoglycemia. For the clinician, CGMS or any other method that continuously provides glucose concentration data will present a challenge to the way we think about hypoglycemia. Instead of an intermittent variable, caregivers will be provided with a continuous variable, analogous to the transition from intermittent blood gas measurements to continuous pulse oximetry for monitoring blood oxygenation. Furthermore, CGMS in this population, as well as in more preterm newborns, will identify numerous episodes of low glucose concentrations that are not identified by intermittent routine blood sampling [41, 42]. In fact, in the populations studied by Harris, et. al., 81 % of all episodes of hypoglycemia were recognized with CGMS only and not by routine clinical blood sampling [42]. There is very little information about the clinical significance of these episodes for long term outcomes, including which should be treated and whether such treatment would improve outcomes. It is possible, therefore, that identifying these episodes of hypoglycemia with CGMS could increase overtreatment of clinically insignificant low glucose concentrations. This risk might outweigh the benefits of documenting more episodes of low glucose concentrations and avoiding treatment of those that resolve spontaneously, as well as the early detection of serious, recurrent hypoglycemia in patients with hyperinsulinemic hypoglycemia and other metabolic disorders [43]. The uncertainty regarding the balance of the risks and benefits of using CGMS or similar devices for the continuous monitoring of neonatal glucose concentrations in this population highlights the need for further studies in this area. Such studies will have to be designed to clarify relationships between continuous glucose concentrations, symptomatic hypoglycemia, responses to treatment, associated medical conditions, and neurodevelopmental outcomes.

## Identifying and treating asymptomatic hypoglycemic newborns

There is very little evidence to inform how often glucose concentrations should be screened in the asymptomatic at-risk newborn or the glucose concentration threshold one should use for treatment. Also unknown are the degree and duration of hypoglycemia necessary to cause permanent neurological injury [9]. Especially important is the question of how to identify the rare infant in which asymptomatic hypoglycemia is the first presentation of a rare and persistent disorder of hypoglycemia such as congenital hyperinsulinemic hypoglycemia, fatty acid oxidation disorders, hypopituitarism, and glycogen storage diseases [8]. In 2011 the AAP published clinical guidelines to address some of these concerns, with special attention to management of hypoglycemia in the first 24 h of life [4]. They suggested that late preterm, LGA, SGA/IUGR, and IDM newborns should be fed by one hour of age and have their glucose checked 30 min after the feeding. Glucose monitoring should then continue before feeds through 12 h of age for LGA and IDM patients as long as pre-feed plasma glucose concentrations remain greater than 40 mg/dL (2.2 mmol/L). It was suggested that late preterm and SGA infants should be screened before feeds for 24 h. In 2012 Harris, et al. published the incidence of hypoglycemia during this same 24 h period, again defining as plasma glucose concentrations <47 mg/dL (2.6 mmol/L) irrespective of age in this same group of newborns [44]. They used a more frequent screening protocol, measuring glucose concentrations one hour after birth and then before feeds every 3–4 h for the first 24 h of life, but also added screening every 3–8 h for the second 24 h of life. While the incidence of hypoglycemia in this population was quite high (51 %), more important were the observations that, of the patients with hypoglycemia, 37 % had their first episode after having had three plasma glucose concentrations greater than 47 mg/dL (2.6 mmol/L) and 6 % had their first episode after 24 h of age [44]. Another important observation in this study was that there were no differences among the at-risk groups studied in the incidence or timing of the hypoglycemia. While the long term clinical significance of these episodes of hypoglycemia, whether first identified in the first 24 h or later, remains unclear, it may be a reasonable approach to simplify screening protocols so that screening frequency and duration are the same for all at-risk groups [44, 45].

The definition of hypoglycemia and the threshold for treatment and continued management are controversial as well. A plasma glucose concentration of 47 mg/dL (2.6 mmol/L) was used in multiple studies by Harris, et al. as their threshold for diagnosis and treatment [32, 34, 36, 42, 44]. The 2011 guideline by the AAP states that once hypoglycemia is identified, treatment should commence with feeding or intravenous dextrose infusion and a target plasma glucose concentration of greater than 45 mg/dL (2.5 mmol/L) should be used [4]. In 2015 the Pediatric Endocrine Society published new recommendations regarding the management of hypoglycemia in newborns [8]. While the main goals of these recommendations were to help clinicians distinguish between physiologically low glucose concentrations in normal newborns and those that persist beyond the first 48 h of life and might place the infant at risk for neurological injury, this group made the recommendation that in the first 48 h of life the target threshold plasma glucose concentration for treatment should be 50 mg/dL (2.8 mmol/L). This group also made the case that neurogenic symptoms occur in newborns below the same glucose concentrations as in adults (55–65 mg/dL [3.1–3.6 mmol/L]) [7, 8]. Thus, they also make the recommendation that in specific patients higher glucose target thresholds of 60 mg/dL (3.3 mmol/L) and 70 mg/dL (3.9 mmol/L) should be used [8]. To date, there is no data to rationally define selection of any one of these lower limit threshold glucose concentration values, in terms of which treatment to use, acute risks vs. benefits, or impact on longer term neurodevelopmental outcomes.

The variability among the recommendations of these different publications reflects the need for further research. A recent publication from the same group that performed the dextrose gel study has provided some data [34], but this was not a study that randomized newborns to different glucose treatment thresholds. However, what they found was that, regardless of treatment with or without the dextrose gel, asymptomatic patients in the main at-risk groups (late preterm, LGA, SGA/IUGR, IDM) had similar neurodevelopmental outcomes at two years of age whether they had hypoglycemia (<47 mg/dL [2.6 mmol/L]) identified in the first seven days of life or not [34]. This assumes that the infants were screened in the first 48 h by the rigorous protocol described above and that when identified, the newborns were treated to achieve or exceed a target plasma glucose concentration of 47 mg/dL (2.6 mmol/L). The main conclusion that one can make from these data is that in this specific patient population with frequent screening and identification of hypoglycemia, using a target plasma glucose concentration of 47 mg/dL (2.6 mmol/L) results in neurodevelopmental outcomes similar to those at-risk patients who did not have an episode of hypoglycemia [34]. However, it cannot be emphasized enough that the plasma glucose concentration defining hypoglycemia was arbitrarily set at 47 mg/dL (2.6 mmol/L) and does not take into account evolution of glucose concentrations and their variability in the first week of life [7]. A plasma glucose concentration of 47 mg/dL (2.6 mmol/L) will likely be less clinically relevant at 6 h of age compared to four days of age, when low glucose concentrations should have increased spontaneously [7].

In the study by McKinlay, et al. the rate of neurodevelopmental delay was not different in the group that experienced a hypoglycemic event (33 %) compared to the group that did not (36 %) [34]. But these rates are high, and some might argue that the threshold for treatment should be even higher than 47 mg/dL (2.6 mmol/L). However, the lack of two year neurodevelopmental outcome data in a concurrently tested group of healthy term control children makes this conclusion premature. It could be that the novel and highly sophisticated techniques that were used to detect abnormal outcomes are so sensitive as to give a high false positive rate and that if a group of healthy term control infants were tested at the same time, they too would have unexpectedly high rates of abnormal outcomes. More importantly, there are significant risks to a more aggressive screening and treatment strategy. These include increased frequency of blood sampling, use of formula supplementation, admission to the NICU and separation from the mother, complications of intravenous catheters, and side effects of any therapies used to raise glucose concentrations. These risks must be balanced against the potential harm due to asymptomatic hypoglycemia, progression to symptomatic hypoglycemia, and delay in diagnosis and treatment of serious metabolic disorders.

The authors also noted three important associations in their data that should provide some caution with respect to increasing glucose treatment thresholds and goals. One is that newborns who did not have a plasma glucose lower than 54 mg/dL (3.0 mmol/L) had worse outcomes than those newborns who did. By CGMS data, the average difference in glucose concentrations between those with worse outcomes and those with better outcomes was only 2.9 mg/dL (0.16 mmol/L). A second important association noted was that those newborns who had more time with plasma glucose concentrations outside the range of 54–72 mg/dL (3.0–4.0 mmol/L) did worse in terms of the highly sensitive neurodevelopmental outcomes than those who had more time within this concentration range. Clearly, these first two associations are in conflict if one were to attempt to change clinical practice based on these observations. Most likely these conflicting associations show that sicker neonates are more metabolically unstable and have worse two year neurodevelopmental outcomes, not that the increased glucose concentration variability necessarily caused the worse outcomes – though this possibility cannot be excluded.

The final important association identified is that, of the hypoglycemic newborns, those with worse outcomes had a steeper rise in their glucose concentrations after treatment with dextrose. This brings up the possibility that in these at-risk asymptomatic newborns with low glucose concentrations, when one decides to commence therapy with intravenous dextrose, it is reasonable to simply start the patient on a continuous glucose infusion rate and not precede this with the traditional 200 mg/kg intravenous dextrose bolus. Previous studies have shown that within 20–30 min hypoglycemic newborns treated without the 200 mg/kg bolus dextrose and only a continuous infusion of dextrose achieve glucose concentrations similar to those treated with the dextrose bolus followed by the same continuous dextrose infusion rate [35]. Given that no study has shown that treatment of asymptomatic hypoglycemia in these patients, no matter how rapid, improves outcomes, it seems that avoiding the steeper rise in glucose concentrations which were associated with worse outcomes in the McKinlay, et al. study is warranted. Other important points to consider when evaluating this study are that severe and symptomatic hypoglycemia was rare. Similarly, hyperglycemia also was rare, as only three patients had a plasma glucose concentration greater than 144 mg/dL (8 mmol/L). It also should be emphasized again that the only patients studied were those who were asymptomatic and late preterm, LGA, SGA/IUGR, and IDM infants [34]. The conclusions, including the abandonment of the 200 mg/kg dextrose bolus, cannot necessarily be extrapolated to other groups, especially those with symptomatic hypoglycemia or a defined or suspected serious metabolic hypoglyyemia disorder, such as congenital hyperinsulinemic hypoglycemia or genetic conditions that lead to excessive glucose utilization (fatty acid oxidation disorders) or insufficient glucose production (hypopituitarism). Nevertheless, abandoning the dextrose bolus might be beneficial in infants of diabetic mothers and those with hyperinsulin-like conditions who respond to rapid increases in glucose concentration with excessive insulin secretion, potentially establishing a rebound hypoglycemia if the continuous dextrose infusion is not high enough.

## Conclusions

Unfortunately, no recently published studies define one ideal strategy to diagnose and appropriately treat potentially damaging low glucose concentrations in neonates. In order to determine the best management strategy, a randomized trial comparing two different strategies with appropriate long term follow up is required, as proposed by Boluyt, et al. in 2006 [46]. Continued use of CGMS as a research tool to demonstrate the impact of different glycemic patterns on long term outcomes in hypoglycemia newborns will be very helpful in this type of study [34, 43]. However, short of this type of study we can look at recently published information and consider two potential changes in how neonatal hypoglycemia is managed (Table 1). One is consideration of dextrose gel as part of a treatment protocol for neonatal hypoglycemia. The second is abandonment of the 200 mg/kg intravenous dextrose bolus for the treatment of asymptomatic, hypoglycemic late preterm, LGA, SGA/IUGR, and IDM newborns.

**Table 1:** Recommendations for the management of neonatal hypoglycemia

1. Buccal dextrose gel should be considered as part of a strategy for managing asymptomatic neonates with low glucose concentrations.
2. For asymptomatic neonates with low glucose concentrations requiring intravenous dextrose, bolus glucose infusions may be replaced by simply starting the patient on a continuous dextrose infusion.
3. Continuous glucose monitoring should be considered in research protocols to assess the benefits and risks of different glycemic patterns for outcomes.

## Abbreviations

CGMS: Continuous glucose monitoring sensor
IDM: Infant of a diabetic mother
IUGR: Intrauterine growth restriction
LGA: Large for gestational age
SGA: Small for gestational age
